# Comparison of the diagnostic efficiency between the O-RADS US risk stratification system and doctors’ subjective judgment

**DOI:** 10.1186/s12880-023-01153-9

**Published:** 2023-11-20

**Authors:** Shan Zhou, Yuyang Guo, Lieming Wen, Jieyu Liu, Yaqian Fu, Fang Xu, Minghui Liu, Baihua Zhao

**Affiliations:** 1 Department of Ultrasound Diagnosis, The Second Xiangya Hospital, Central South University, No.139, Renmin Middle Road, Changsha, 410011 Hunan China; 2 Health Management Center, The Second Xiangya Hospital, Central South University, No.139, Renmin Middle Road, Changsha, 410011 Hunan China; 3 Department of Ultrasonography, The First Hospital of Changsha, No.311, Yingpan Road, Changsha, 410005 Hunan China

**Keywords:** O-RADS, Adnexal mass, Diagnostic efficiency, Pelvic ultrasound

## Abstract

**Background:**

This study aimed to compare the diagnostic efficiency of Ovarian-Adnexal Reporting and Data System (O-RADS) and doctors’ subjective judgment in diagnosing the malignancy risk of adnexal masses.

**Methods:**

This was an analysis of 616 adnexal masses between 2017 and 2020. The clinical findings, preoperative ultrasound images, and pathological diagnosis were recorded. Each adnexal mass was evaluated by doctors’ subjective judgment and O-RADS by two senior doctors and two junior doctors. A mass with an O-RADS grade of 1 to 3 was a benign tumor, and a mass with an O-RADS grade of 4–5 was a malignant tumor. All outcomes were compared with the pathological diagnosis.

**Results:**

Of the 616 adnexal masses, 469 (76.1%) were benign, and 147 (23.9%) were malignant. There was no difference between the area under the curve of O-RADS and the subjective judgment for junior doctors (0.83 (95% CI: 0.79–0.87) vs. 0.79 (95% CI: 0.76–0.83), *p* = 0.0888). The areas under the curve of O-RADS and subjective judgment were equal for senior doctors (0.86 (95% CI: 0.83–0.89) vs. 0.86 (95% CI: 0.83–0.90), *p* = 0.8904). O-RADS had much higher sensitivity than the subjective judgment in detecting malignant tumors for junior doctors (84.4% vs. 70.1%) and senior doctors (91.2% vs. 81.0%). In the subgroup analysis for detecting the main benign lesions of the mature cystic teratoma and ovarian endometriosic cyst, the junior doctors’ diagnostic accuracy was obviously worse than the senior doctors’ on using O-RADS.

**Conclusions:**

O-RADS had excellent performance in predicting malignant adnexal masses. It could compensate for the lack of experience of junior doctors to a certain extent. Better performance in discriminating various benign lesions should be expected with some complement.

## Background

Ovarian cancer is the fifth most common cause of cancer death in women, with a general survival rate of < 50% [1]. Detection and diagnosis of adnexal mass (AM) still face severe challenges. Due to its low cost and accessibility, ultrasound is the first-choice modality to detect AMs and estimate the malignancy risk. However, the AMs originating from different tissues often have various sonographic findings [2], so the ultrasound diagnosis is highly experience-dependent. So far, the subjective judgment of experienced gynecological ultrasound experts is considered to be the most valuable means to diagnose benign and malignant AMs [3]. Due to the lack of diagnostic tools with high accuracy and consistent interpretations, junior doctors need much practice over a long period to achieve excellent diagnostic performance.

Since the 1990s, many ultrasound systems for AMs have been proposed and improved [4–12]. In recent years, the reliability and efficiency of the International Ovarian Tumor Analysis (IOTA) rules, the Risk of Malignancy Index (RMI) and the Gynecologic Imaging Reporting and Data System (GI-RADS) have been validated by many studies and in different ethnic groups [4–12]. To some extent, these predictive models can improve the diagnostic accuracy of junior doctors. However, the descriptions and definitions of malignant signs vary and are confusing.

In 2018, the Ovarian-Adnexal Reporting and Data System (O-RADS) US working group published a white paper to describe the standardized lexicon for ovarian and adnexal lesions to improve the quality and communication of imaging reports between ultrasound examiners and referring clinicians [13]. In 2020, the O-RADS US risk stratification and management system (O-RADS system) was proposed by the American College of Radiology based on the white paper [13]. It was designed to provide consistent interpretations and appropriate management and reach a higher probability of accuracy in assigning malignancy risk to AMs. O-RADS is the only lexicon and classification system of ovarian lesions. The recommended six categories (O-RADS 0–5) encompass the range from normal to high risk of malignancy [11, 14–17].

Since the validated data were based on a European population and the evaluations of some categories in the O-RADS depended on doctors’ diagnostic experience, large interobserver variability studies in other ethnic groups are needed to validate the use of the system by expert and less experienced observers. This study aimed to compare the diagnostic efficiency of O-RADS and doctors’ subjective judgment in predicting the malignancy of AMs in the East Asian population and the consistencies in the application of the system by doctors with different diagnostic experiences.

## Methods

### Study sample

This was an analysis of 599 women who underwent gynecological surgery in the Second Xiangya Hospital between June 2017 and June 2020. The clinical records, diagnostic ultrasound images, and pathological findings were collected from the workstation. Patients over 50 years old who underwent hysterectomy were regarded as postmenopausal.

We included women based on the following criteria: 1) the interval between ultrasound and gynecological surgery was less than 30 days; 2) definite histopathological findings and 3) a lesion with more than one representative image for recognizing the ultrasound features. We excluded pregnant women with pelvic masses. Patients with only ascitic fluid cytology results were not included.

**Fig. 1:**
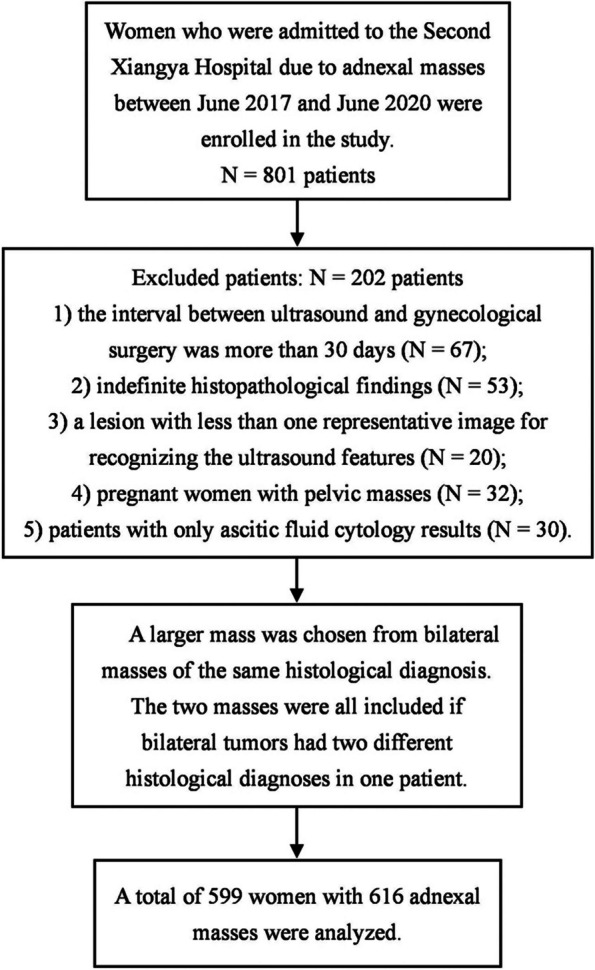
Flow chart of patient enrollment

The images were acquired by ultrasound diagnostic systems with a 9–15 MHz intracavitary transducer. We performed a transabdominal ultrasound if the lesion was too large to observe using transvaginal ultrasound. The image acquisition and lesion description were carried out in accordance with the expert consensus of the IOTA Working Group on the description of the sonographic features of AMs [9]. The static images showed the lesions in multiple sections and angles and included all key sonographic features of the lesions as single or bilateral, lesion echo (cystic, solid or solid-cystic), the maximum diameter of the lesion and the solid part of the lesion, morphology (regular or irregular), margins (smooth or unsmooth), cyst wall (smooth or irregular) and thickness, cyst content, calcification component, acoustic shadowing, solid papillary protrusions, septum, ascites, peritoneal or pelvic wall nodules, and color Doppler score. All images were stored and collected later from the ultrasound workstation. A larger mass was chosen from bilateral masses of the same histological diagnosis. The two masses were all included if bilateral tumors had two different histological diagnoses in one patient (Fig. 1).

### Ethics approval

The data collection was approved by the Medical Ethics Committee of Second Xiangya Hospital of Central South University (No. 2021–38) and conducted according to the principles of the Declaration of Helsinki. The need for the patient’s informed consent was waived by the Medical Ethics Committee of Second Xiangya Hospital of Central South University. No personal privacy was released.

### Image analysis with O-RADS

Wen and Zhao (Group I) are senior ultrasonic doctors with more than 10 years of working experience, and they are also experts in the gynecological ultrasound medicine [18]. Guo and Zhou (Group II) are junior ultrasonic doctors with 1 year of working experience and diagnosis practice of 300 adnexal tumors. A test-retest series of 40 AMs was performed to test the intraclass agreement.

Each doctor first diagnosed the 40 AMs by subjective judgment. The κ value was 0.86 (95% CI: 0.63–1.00) for Group I and 0.81 (95% CI: 0.58–1.00) for Group II. Subsequently, the doctors of the two groups made subjective judgments on all AMs and recorded the benign or malignant results.

One month after subjective judgment, the four doctors received theoretical and practical training for O-RADS. Then, each doctor used O-RADS to diagnose the 40 randomly selected masses. The weighted κ value was 0.92 (95% CI: 0.78–1.00) for Group I and 0.93 (95% CI: 0.81–1.00) for Group II. Finally, the doctors described the ultrasound features in a standard manner according to the O-RADS ultrasound lexicon. The two groups analyzed all images and graded each AM using O-RADS. In each group, the two doctors cooperated in analyzing the images. Masses of O-RADS grades of 1–3 were identified as benign lesions, and masses of O-RADS grades of 4–5 were identified as malignant tumors [14]. Doctors in both groups were not involved in the data collection process. All analyses were blinded to the clinical data and histopathological results.

We compared all results with the pathological diagnosis based on the International Federation of Gynecology and Obstetrics criteria and the World Health Organization International Classification of Ovarian Tumors [19, 20], and borderline AMs were considered as malignant.

Except for ultrasound diagnosis, the doctors who evaluated the AMs before the surgery also included radiologists skilled in gynaecological imaging diagnosis and gynaecologists in our hospital. And there are dedicated pathologists in our hospital to complete the pathological diagnosis of the AMs after the surgery. They are all experts in the pathology of ovarian-adnexal tumors, with 10 years or more of diagnostic experience.

### Statistical analysis

We used the SPSS version 26.0 (SPSS Inc., Chicago, IL, USA), GraphPad Prism version 6.0 (GraphPad Software Inc., San Diego, CA, USA) and MedCalc Statistical Software version 19.0.7 (MedCalc Software bvba, Ostend, Belgium) to perform statistical analysis. The chi-square test or Fisher’s exact test was applied to compare categorical variables. Shapiro-Wilk test was used to test whether the continuous variables were normally distributed. The normally distributed continuous variables were described by the mean ± standard deviation and compared by the independent samples t-test. The non-normally distributed continuous variables were described by the median (quartile spacing) and compared by the rank sum test. McNemar’s test was used for comparing Dichotomous paired data. The diagnostic performance of the two methods was tested by using receiver operating characteristic (ROC) analysis, and the comparison of areas under curve (AUCs) was done using MedCalc software. The intergroup agreement between the junior and senior doctors was tested by the kappa coefficient. High repeatability was indicated by a κ ≥ 0.75, medium repeatability was indicated by an of 0.40 ≤ *κ* < 0.75, and low repeatability was indicated by a *κ* < 0.40. A significant statistical difference was represented by a *p* < 0.05.

## Results

A total of 616 AMs that were 469 (76.1%) benign and 147 (23.9%) malignant were analyzed. Teratoma (217/469, 46.2%) was the most common benign adnexal tumor and adenocarcinoma (57/147, 38.8%) was the most common malignant adnexal tumor in histopathological findings (Table 1).

**Table 1:** Histopathological diagnosis of 616 adnexal masses

Histopathology	N (%)
**Benign lesion**	**469 (76.1)**
Mature cystic teratoma	217 (35.2)
Ovarian endometriosic cysts	75 (12.2)
Serous cystadenoma and Mucinous cystadenoma	66 (10.7)
Adnexal inflammatory mass	44 (7.1)
Simple cyst	29 (4.7)
Corpus luteum	6 (1.0)
Benign mixture ovarian tumor	6 (1.0)
Other benign tumors	26 (4.2)
**Malignant lesion**	**147 (23.9)**
Adenocarcinoma	57 (9.3)
Serous cystadenocarcinoma and Mucinous cystadenocarcinoma	25 (4.1)
Borderline cystadenoma	21 (3.4)
Granulosa cell tumor	11 (1.8)
Endometrioid adenocarcinoma	6 (1.0)
Yolk sac tumor	6 (1.0)
Immature teratoma and Malignant transformation of mature cystic teratoma	5 (0.8)
Clear cell tumor	4 (0.6)
Malignant mixture ovarian tumor	3 (0.5)
Borderline brenner tumor	1 (0.2)
Other malignant tumors	8 (1.3)

The clinical characteristics are shown in Table 2. Of the 599 women, bilateral masses were found in 123(20.5%) women, including 17(2.8%) with two different tumors. Malignant tumors were found in much older women with higher CA125 levels, and more patients were in the postmenopausal period. Patients with malignant lesions presented more bilateral masses.

**Table 2:** Demographic and clinical characteristics of 599 women with 616 masses

Characteristics		Women with benign lesions (*n* = 455)	Women with malignant lesions (*n* = 144)	P^*^
Age (years)		26.0 (26.0, 46.0)	49.0 (38.0, 54.0)	< 0.001^**^
Postmenopause		70 (15.4)	64 (44.4)	< 0.001
Premenopause		385 (84.6)	80 (55.6)	
Bilateral lesion		75 (16.5)	48 (33.3)	< 0.001
Unilateral lesion		380 (83.5)	96 (66.7)	
CA125	Increased	81 (17.8)	105 (72.9)	< 0.001
	Normal	355 (78.0)	38 (26.4)	
	No record	19 (4.2)	1 (0.7)	

Values are shown as median (the first quartile, the third quartile) or numbers (%)

CA125: cancer antigen 125

*: Chi-square test

**: Wilcoxon test

By subjective judgment, 456 (74.0%) AMs were classified as benign lesions and 160 (26.0%) AMs were classified as malignant tumors in Group I. 460 (74.7%) AMs were classified as benign lesions and 156 (25.3%) AMs were classified as malignant tumors in Group II.

**Fig. 2:**
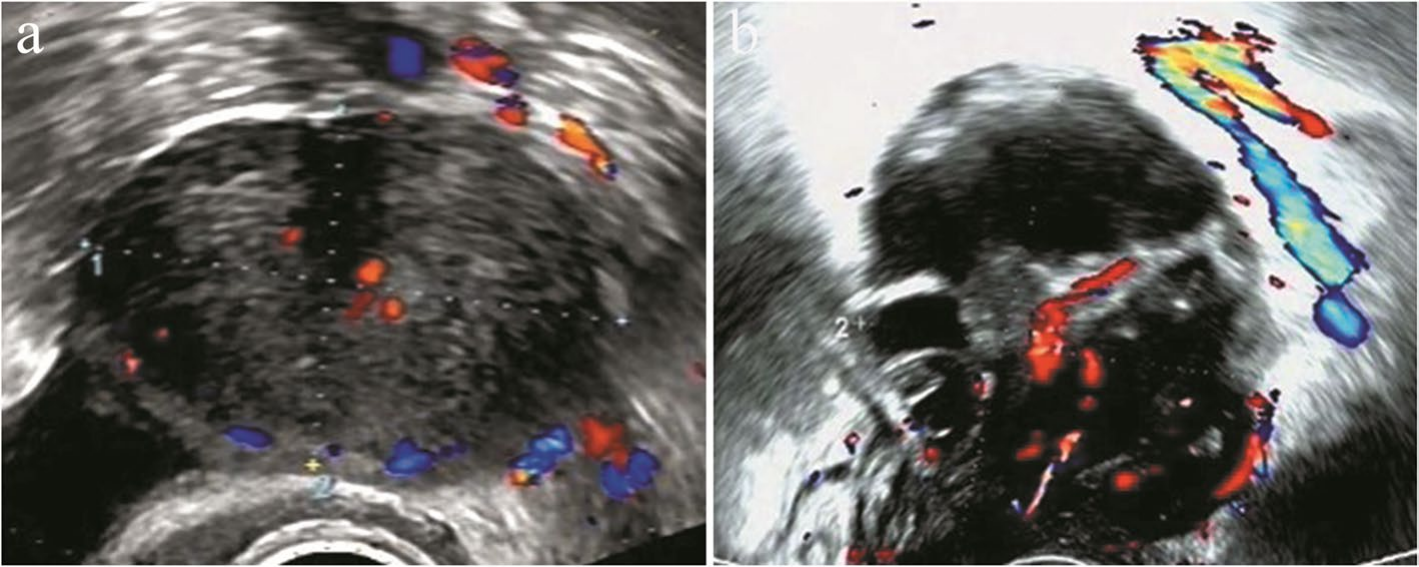
Ultrasound images supporting the difference in results between the groups. **a**. Image of a pathologically proven adult granulosa cell tumor from a patient (case 172 among the 599 women) is shown. This was a smooth solid mass with a color score of 2–3. Both senior and junior doctors classified the mass as O-RADS 4. But Senior doctors subjectively diagnosed it as malignant, while junior doctors subjectively diagnosed it as benign. **b**. Image of a pathologically proven mucinous cystadenoma from a patient (case 204 among the 599 women) is shown. This was a multilocular cyst with solid component, and with a color score of 3–4. Senior doctors classified the mass as O-RADS 5, and junior doctors classified it as O-RADS 4. Both senior and junior doctors diagnosed the mass as malignant by subjective judgment

In Group I, 395 (64.1%) AMs were classified as benign lesions: 1 case of O-RADS grade 1, 313 cases of O-RADS grade 2, and 81 cases of O-RADS grade 3. A total of 221 (35.9%) masses were classified as malignant tumors: 161 cases of O-RADS grade 4 and 60 cases of O-RADS grade 5. No (0/1) histological malignant tumors were O-RADS grade 1, 1.9% (6/313) were O-RADS grade 2, 8.6% (7/81) were O-RADS grade 3, 50.9% (82/161) were O-RADS grade 4, and 86.7% (52/60) were O-RADS grade 5. In Group II, 403 (65.4%) benign lesions were diagnosed as O-RADS grades 1 to 3, and 213 (34.6%) malignant tumors were diagnosed as O-RADS grades 4 to 5. The malignancy rates of O-RADS grades 1 to 5 were 0.0% (0/6), 2.7% (8/292), 14.3% (15/105), 40.7% (48/118) and 80.0% (76/95), respectively (Fig. 2). Table 3 displays the ultrasonographic descriptions of the masses.

**Table 3:** Ultrasonographic characteristics of the masses

Ultrasonographic characteristic		Ultrasound images	Pathological results
Simple unilocular cyst, without solid component			Simple cyst
Unilocular cyst, with irregular inner wall (< 3 mm height)			Borderline serous papillary cystadenoma
Unilocular cyst, with solid component (≥ 3 mm height) ^a^			Endometrioid adenocarcinoma
Multilocular cyst, without solid component (≥ 3 mm height) ^a^			mucinous cystadenoma
Multilocular cyst, with solid component (≥ 3 mm height) ^a^			adenocarcinoma
Solid lesion, with smooth outer contour			struma-ovarii
Solid lesion, with irregular outer contour			serous carcinoma
Classic Benign Lesions	Not simple, unilocular cyst, with thick wall and without solid component		corpus luteum cyst
	Not simple, unilocular cyst, with opaque echo within the cyst and without solid component		ovarian endometriosic cyst
	Not simple, unilocular cyst, with high echo clumps or strong echo spots within the cyst and without solid component		mature cystic teratoma
	Strip shaped cyst with incomplete separation		hydrosalpinx

^a^solid component includes a papillary projection or solid component that is not a papillary projection

**Fig. 3:**
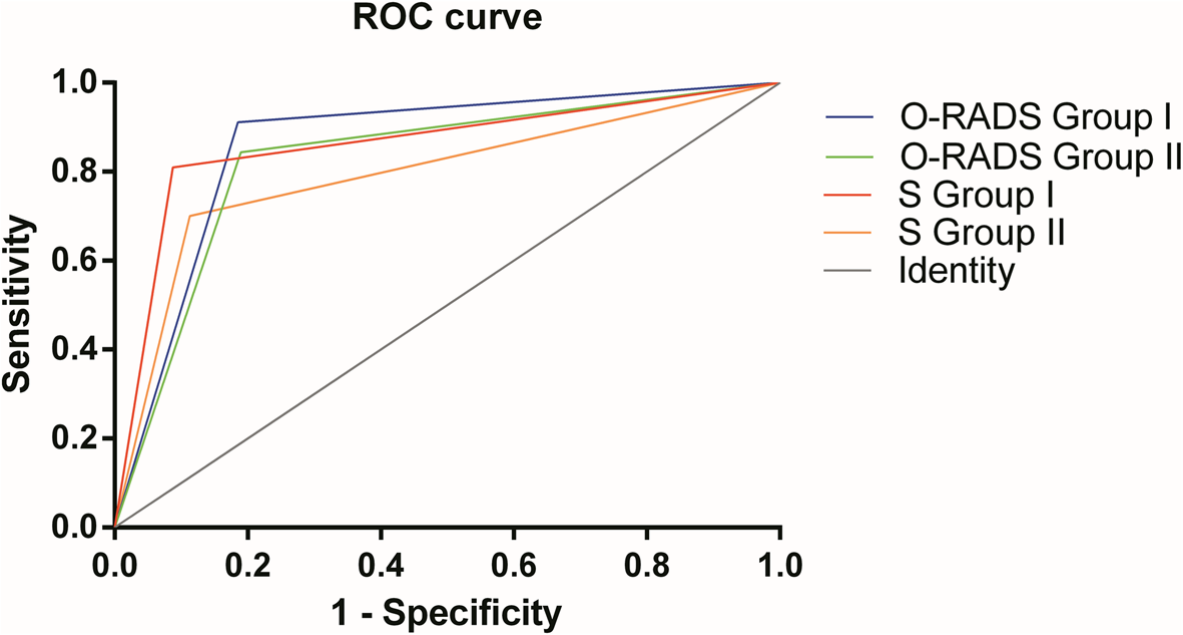
ROCs for O-RADS and the subjective judgment of the two groups. O-RADS: Ovarian Adnexal Reporting and Data System; S: subjective judgment

The ROC curves for O-RADS and subjective judgment in predicting malignant tumors are shown in Fig. 3. The area under the curve (AUC), sensitivity, specificity, negative predictive value (NPV), and positive predictive value (PPV) for the two groups in classifying the 616 AMs using O-RADS and doctors’ subjective judgment are shown in Table 4. There was no difference between the area under the curve (AUC) of O-RADS and the subjective judgment for Group II (0.83 vs. 0.79, *p* = 0.0888). The AUCs of O-RADS and subjective judgment were equal for Group I (0.86 vs. 0.86, *p* = 0.8904). The AUC of O-RADS in Group I was higher than that in Group II (0.86 vs. 0.83, *p* = 0.0183). In the two groups, O-RADS had much higher sensitivity but much lower specificity than subjective judgment in detecting malignant tumors.

**Table 4:** The diagnostic accuracy of the two methods in predicting the malignant adnexal masses (*n* = 616)

Group	Method	Sens.	Spec.	PPV	NPV	AUC (95%CI)
Group I	Subjective judgment	81.0%	91.3%	74.4%	93.9%	0.86 (0.83–0.89)
	O-RADS grading	91.2%	81.4%	60.6%	96.7%	0.86 (0.83–0.90)
Group II	Subjective judgment	70.1%	88.7%	66.0%	90.4%	0.79 (0.76–0.83)
	O-RADS grading	84.4%	81.0%	58.2%	94.3%	0.83 (0.79–0.87)

*Sens.* sensitivity, *Spec.* specificity, *PPV* Positive predictive value, *NPV* Negative predictive value, *AUC* Area under receiver-operator characteristics curve, *CI* Confidence interval

**Fig. 4:**
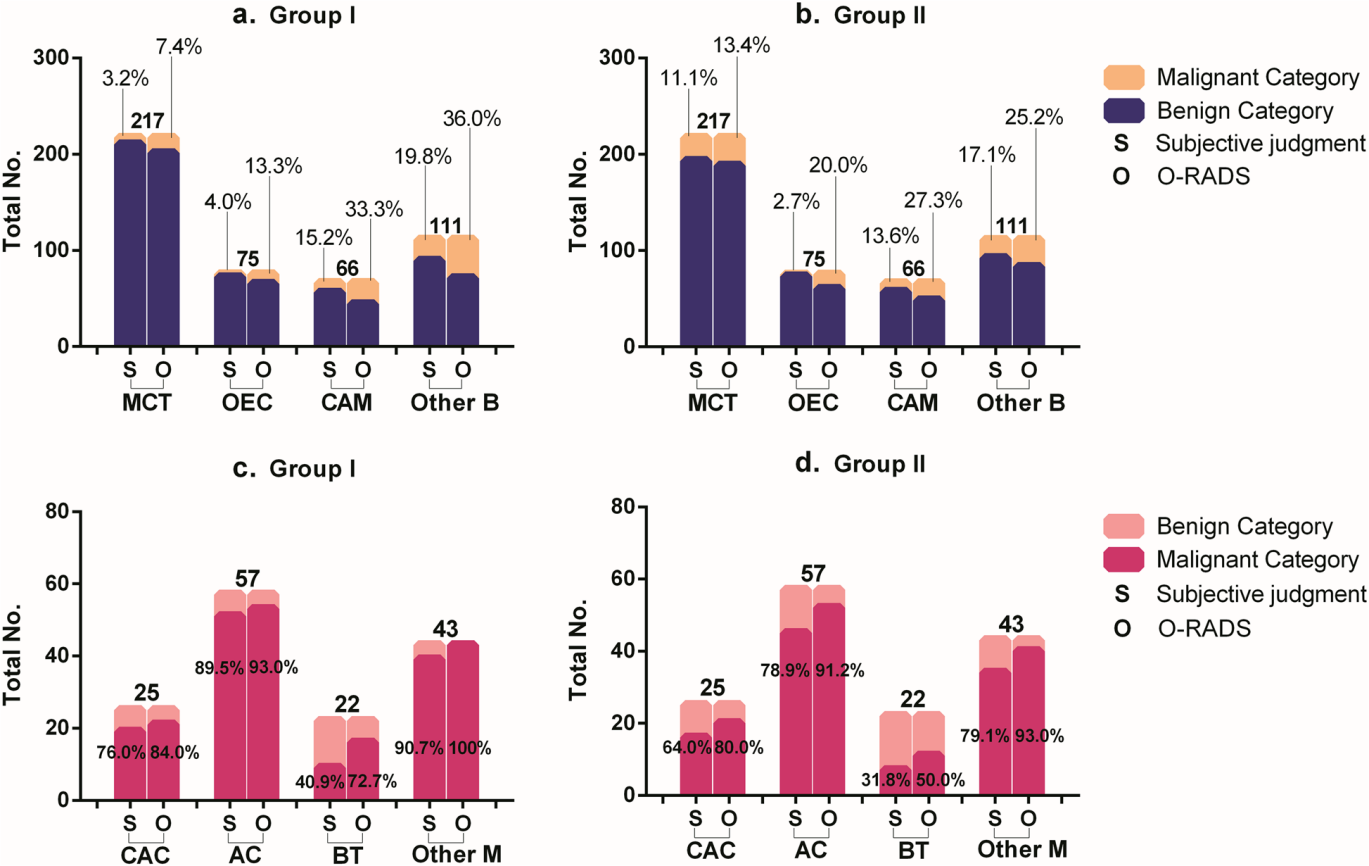
Frequency distributions of AMs in different pathological classifications of O-RADS and subjective judgment. **a**. The inaccuracy of using O-RADS and subjective judgment in common pathological benign lesions in group I; **b**. The inaccuracy of using O-RADS and subjective judgment in common pathological benign lesions in group II; **c**. The accuracy of using O-RADS and subjective judgment in common pathological malignant tumors in group I; **d**. The accuracy of using O-RADS and subjective judgment in common pathological malignant tumors in group II. O-RADS: Ovarian Adnexal Reporting and Data System; MCT: mature cystic teratoma; OEC: ovarian endometriosic cyst; CAM: cystadenoma; Other B: other benign lesions; CAC: cystadenocarcinoma; AC: adenocarcinoma; BT: borderline tumors; Other M: other malignant tumors

On subgroup analysis, for detecting the main benign lesions of the mature cystic teratoma (217/616, 35.2%) and ovarian endometriosic cyst (75/616, 12.2%), the junior doctors’ diagnostic accuracy was obviously worse than the senior doctors’ when using O-RADS (Table 5). In predicting each kind of malignant tumor, O-RADS had much higher accuracy than doctors’ subjective judgment, especially for junior doctors. It had quite lower accuracy in diagnosing all kinds of benign lesions (Fig. 4).

**Table 5:** The diagnostic performance for assessing the most common benign lesions and malignant tumors

Histologic diagnosis	No. (%)	Diagnostic accuracy of O-RADS system		Diagnostic accuracy of Subjective judgment	
		Group I	Group II	Group I	Group II
Benign mass					
Mature cystic teratoma	217 (35.2)	201 (92.6)	188 (86.6)	210 (96.8)	193 (88.9)
Ovarian endometriosic cysts	75 (12.2)	65 (86.7)	60 (80.0)	72 (96.0)	73 (97.3)
Cystadenoma	66 (10.7)	45 (68.2)	49 (74.2)	57 (86.4)	58 (87.9)
Other benign lesions	111 (18.0)	71 (64.0)	83 (74.8)	89 (80.2)	92 (82.9)
Malignant mass					
Cystadenocarcinoma	25 (4.1)	22 (88.0)	20 (80.0)	20 (80.0)	17 (68.0)
Adenocarcinoma	57 (9.2)	53 (93.0)	52 (91.2)	51 (89.5)	45 (78.9)
Borderline tumors	22 (3.6)	16 (72.7)	11 (50.0)	9 (40.9)	8 (36.4)
Other malignant tumors	43 (7.0)	43 (100)	41 (95.3)	39 (90.7)	33 (76.7)

Values are shown as numbers (%)

*No.* Number, *O-RADS* Ovarian Adnexal Reporting and Data System

For the diagnosis of benign and malignant tumors, the intergroup agreement between the two groups was good for O-RADS (κ = 0.70, 95% CI: 0.64–0.76, *p* < 0.001) and the subjective judgment (κ = 0.67, 95% CI: 0.60–0.73, *p* < 0.001).

## Discussion

The diagnosis of ovarian cancer is often delayed because of its complex tissue origin and lack of specific clinical manifestations and biological markers in the early stage of the disease. More than 75% of patients are diagnosed in the late stage of ovarian cancer. Accurate screening and diagnosis of ovarian cancer are still challenges in clinics [21].

Ultrasound has always been the preferred examination method for diagnosing benign and malignant ovarian tumors. Considering the early and accurate diagnosis of ovarian malignancies and that some young women undergoing surgery must maintain fertility, the preoperative and postoperative follow-up accurate ultrasound diagnosis is particularly important. In clinical work, the subjective judgment of ultrasound doctors cannot directly diagnose borderline ovarian tumors (BOTs), and gynecologists attach great importance to protecting the reproductive function of young BOT patients. During preoperative and postoperative ultrasound examination, it is crucial to sensitively detect any suspicious signs of disease progression, so that gynecologists can make timely and correct clinical decisions [22, 23].

The imaging findings of ovarian tumors are complex and diverse. The benign, borderline and malignant ovarian tumors with overlapping imaging features always lack specific imaging features [24–26]. Even experienced gynecological ultrasound experts can only infer the possible source of ovarian tumors (such as epithelial or sex cord stromal sources) and the possibility of benign or malignant tumors by their subjective judgment. Ultrasound doctors have been committed to improving the diagnostic accuracy of the benign and malignant AMs. Their summaries of the specific ultrasound features and malignant features of AMs, as well as the reporting of special cases of AMs, have been ongoing for many years and have been constantly improving [27–29]. Although ultrasound diagnostic experts are constantly trying to identify and summarize more subtle ultrasound manifestations of ovarian tumors that are prone to confusion [30–32], these methods still require further clinical validation.

In this context, some grading systems or prediction models, such as IOTA SR, IOTA LR2, IOTA SRRA, ADNEX, RMI4, and GI-RADS, have been developed to predict the malignancy of AMs to improve the diagnostic accuracy for malignant tumors [8, 12, 21, 33, 34]. These systems and models summarized the key ultrasound features of ovarian tumors through large-sample research and have undergone many prospective or retrospective external validations. It is hoped that they can sensitively and accurately detect ovarian tumors with malignant risk, and thus guide clinical strategy more accurately and objectively. To some extent, these diagnostic models could compensate for junior doctors’ inexperience in predicting the malignancy of AMs.

We performed an external validation of O-RADS, which was the only system that provided a detailed characteristic explanation and description of a large part of benign and malignant lesions to ensure the highest sensitivity in detecting malignant masses. This point was well validated in the analysis of the 616 masses by senior and junior doctors. O-RADS had higher sensitivity in detecting all kinds of malignant tumors than the junior doctors’ subjective judgment (83.7% vs. 70.1%) and the senior doctors’ diagnosis (91.2% vs. 81.0%). It could identify the actual malignancy of the lesion to the greatest extent possible, reducing the serious consequences associated with missed diagnoses. This indicates the vital capability of a malignant tumor predictive model. Patients with highly suspected malignant lesions should be advised to consult a gynecologic oncologist and treated timely.

There is no doubt that higher sensitivity was achieved at the cost of decreased specificity. Another essential advantage of this system is that it proposes management recommendations for patients with each grade of malignancy risk. Subsequent examinations and clinical measures have been advised for these patients [14]. Patients with suspected malignant lesions should be arranged for a magnetic resonance imaging examination or consultation with an ultrasound expert. Experts may correct some misdiagnoses since senior doctors performed better in detecting all kinds of benign tumors than this system in our study.

The two groups of doctors achieved medium intergroup agreement when applying O-RADS, and the AUCs of O-RADS and subjective judgment had no differences in the two groups. This showed that doctors’ experience had an impact on the diagnostic efficiency of the O-RADS system to a certain extent. Compared with diagnosing other kinds of tumors using O-RADS, junior doctors performed much worse than senior doctors diagnosing classical benign lesions in our study. Similar to other studies, common benign tumors of ovarian endometriosic cysts and inflammatory masses accounted for 19.3% of our study sample. Endometriosis tended to occur in women of childbearing age, accounting for approximately 35% of benign pelvic tumors, severely affecting fertility [35, 36], and relating to malignant degeneration of ovarian lesions [37]. Accurately diagnosing and properly managing them as early as possible may protect their fertility. However, the diagnosis of these classical benign lesions depends largely on doctors’ subjective judgment. Thus, an ovarian tumor diagnostic system should provide more detailed diagnostic characteristics to help junior doctors discriminate lesions from malignant tumors and other classical benign lesions.

There were many limitations that should be acknowledged in this study. First, Dynamic images could not be obtained for evaluation. Second, the low malignancy rate (23.9%) in our study sample may be one of the causes of the lower diagnostic specificity of O-RADS. Third, the performance of the following management recommendations needs to be further evaluated by more prospective studies.

## Conclusions

O-RADS had excellent performance in predicting malignant AMs. Doctors’ experience has an impact on the diagnostic efficiency of the system. O-RADS could compensate for the lack of experience of junior doctors to a certain extent for its high sensitivity. Better performance in discriminating various benign lesions should be expected with some complement.

## Data Availability

The datasets used during the current study are available from the corresponding author upon reasonable request.

## Abbreviations

AM: Adnexal mass
IOTA: International Ovarian Tumor Analysis
RMI: Risk of Malignancy Index
GI-RADS: Gynecologic Imaging Reporting and Data System
O-RADS: Ovarian-Adnexal Reporting and Data System
ROC: Receiver operating characteristic
AUC: Area under curve
PPV: Positive predictive value
NPV: Negative predictive value
